# Assessment of Somatosensory Function and Self-harm in Adolescents

**DOI:** 10.1001/jamanetworkopen.2021.16853

**Published:** 2021-07-13

**Authors:** Tatum M. Cummins, Oliver English, Helen Minnis, Daniel Stahl, Rory C. O’Connor, Kirsty Bannister, Stephen B. McMahon, Dennis Ougrin

**Affiliations:** 1 Neurorestoration, Institute of Psychiatry, Psychology, and Neuroscience, King’s College London, London, United Kingdom; 2 Central Modulation of Pain, Institute of Psychiatry, Psychology, and Neuroscience, King’s College London, London, United Kingdom; 3 Child and Adolescent Psychiatry, Institute of Psychiatry, Psychology, and Neuroscience, King's College London, London, United Kingdom; 4 Adverse Childhood Experiences Clinical and Research Centre, Institute of Health and Wellbeing, University of Glasgow, Glasgow, United Kingdom; 5 Biostatistics and Health Informatics, Institute of Psychiatry, Psychology, and Neuroscience, King's College London, London, United Kingdom; 6 Suicidal Behaviour Research Laboratory, Institute of Health and Wellbeing, University of Glasgow, Glasgow, United Kingdom

## Abstract

**Importance:**

Self-harm is a risk factor for suicide in adolescents, with the prevalence highest in young people in group and residential care programs. Although no established risk factors for self-harm exist, adolescents who self-harm may have decreased pain sensitivity, but this has not been systematically investigated.

**Objective:**

To assess somatosensory function using quantitative sensory testing (QST) in children and adolescents living in care grouped by the number of episodes of self-harm in the past year and compare their somatosensory profiles with community control participants to investigate associations with the incidence or frequency of self-harm.

**Design, Setting, and Participants:**

Recruitment for this cross-sectional study began January 2019 and ended March 2020. Exclusion criteria included intellectual disability (intelligence quotient <70), autism spectrum disorder, or recent serious injury. Children and adolescents aged 12 to 17 years with no underlying health conditions were recruited from local authority residential care settings in Glasgow, UK, and schools and youth groups in London and Glasgow, UK. The volunteer sample of 64 participants included adolescents ages 13 to 17 years (34 [53%] females; 50 [78%] living in residential care; mean [SD] age, 16.34 [1.01] years) with varying incidents of self-harm in the past year (no episodes, 31 [48%]; 1-4 episodes, 12 [19%]; and ≥5 episodes, 2 [33%]).

**Exposures:**

Participants were tested using a standardized QST protocol to establish baseline somatosensory function.

**Main Outcomes and Measures:**

Associations between somatosensory sensitivity, incidence and frequency of self-harm, residential status, age, gender, and prescription medication were calculated. Secondary outcomes assessed whether self-harm was associated with specific types of tests (ie, painful or nonpainful).

**Results:**

A total of 64 participants ages 13 to 17 years completed testing (mean [SD] age, 16.3 [1.0] years; 34 [53%.] females and 30 [47%] males; 50 [78%] living in group homes). Adolescents with 5 or more self-harm incidences showed significant pain hyposensitivity compared with community control participants after adjusting for age, gender, and prescription drug use (SH group with 5 or more episodes vs control: −1.03 [95% CI, −1.47 to −0.60]; *P* < .001). Hyposensitivity also extended to nonpainful stimuli, similarly adjusted (SH group with 5 or more episodes vs control: −1.73; 95% CI, −2.62 to −0.84; *P* < .001). Pressure pain threshold accounted for most of the observed variance (31.1% [95% CI, 10.5% to 44.7%]; *P* < .001).

**Conclusions and Relevance:**

The findings of this study suggest that sensory hyposensitivity is a phenotype of Adolescents who self-harm and that pressure pain threshold has clinical potential as a quick, inexpensive, and easily interpreted test to identify adolescents at increased risk of repeated self-harm.

## Key Points

**Question:**

Is pain sensitivity associated with self-harm frequency in children and adolescents aged 12 to 17 years?

**Findings:**

This cross-sectional study of 64 adolescents systematically examined sensitivity to a range of painful and nonpainful sensory stimuli. Pain hyposensitivity was significantly associated with self-harm frequency, and these findings also extended to nonpainful sensory stimuli; pressure pain threshold was associated with significant specificity and sensitivity for incidence of self-harm.

**Meaning:**

These findings suggest that pressure pain threshold is a novel biomarker for identifying adolescents at high risk of self-harm.

## Teaser

This cross-sectional study assesses somatosensory function in adolescents and compares their somatosensory profiles with community control participants to investigate associations with the incidence and frequency of self-harm.

## Introduction

Suicide is the second leading cause of death among adolescents in most Western countries.^1^ Self-harm necessitating hospital treatment is the strongest known predictor of suicide.^2^ Both suicide and self-harm in adolescents have been rising in the UK and elsewhere.^3^ The UK and other European countries define self-harm as self-poisoning or self-injury irrespective of the suicidal intent,^4^ with self-injury being the most prevalent in community samples.^5^ Typical onset of self-harm is in adolescents aged 12 to 14 years and is higher in girls and individuals with experience of childhood maltreatment; prevalence is estimated to be 10% and 25% in community samples.^6,7,8,9,10^ Young people living in local authority-run group homes and residential care programs because of maltreatment are particularly high risk for self-harm and suicide.^11^ Young people living in care constitute less than 1% of the UK population aged younger than 18 years, yet they account for about half of all suicides in the UK.^12^

The World Health Organization (WHO) has highlighted the need for identifiable risk and protective factors in suicide prevention,^13^ but decades of research have failed to produce robust and specific risk factors that identify individuals at higher risk of self-harm or suicide.^14,15^ Advances have been made using machine learning and functional imaging to identify adolescents who are suicidal,^16,17^ but there are few recognized biological, clinical, or psychological risk factors that could be applied clinically with confidence.^18^

Contemporary theories of suicidal behavior^19,20,21^ posit that individuals who engage in suicidal behavior must develop an acquired capability to do so; a component of this is proposed to be an elevated pain tolerance. There is some evidence that individuals who self-harm have altered pain perception, but the causes remain obscure, and the impact of self-harm on nonpain somatosensory sensitivity is even less well understood.^22,23,24,25^ Childhood maltreatment increases the risk of self-harm and suicide attempts^26,27^ and is strongly linked to aberrant pain perception.^28^ A seminal meta-analysis showed self-harm to be significantly associated with higher pain thresholds.^29^ However, it remains unclear whether pain sensitivity differences are a consequence of self-harm, an effect of psychiatric comorbidity, or whether these differences are present prior to onset of self-harm.

The present study focused on answering 3 questions. First, does pain sensitivity differ between young people in residential care with and without self-harm and community-dwelling control participants? Second, do these perceptual differences extend to nonpainful stimuli? Third, is pain sensitivity associated with frequency of self-harm? Finally, we systematically investigated whether 1 or more sensory tests could be used as a simple yet specific clinical biomarker to identify adolescents at high risk of self-harm.

## Methods

This cross-sectional study was approved by the University of Glasgow College of Medical, Veterinary, and Life Sciences and King’s College London research ethics committees. Written informed consent was obtained from participants aged 16 years or older or from parents or caregivers for participants younger than 16 years. We followed Strengthening the Reporting of Observational Studies in Epidemiology (STROBE) reporting guideline for cross-sectional studies.

### Participants

Adolescents aged 12 to 17 years with no underlying health conditions were recruited from both the London and Glasgow area via schools, Child and Adolescent Mental Health Services, secure and residential care homes, and after school clubs. Social work staff attached to young people in residential and foster care in Glasgow identified young people with self-harm and approached them directly with information about the study. The research team also had help identifying young people in residential care with self-harm via collaborative sites. Professionals (eg, psychiatrist, psychologist, unit manager, or teacher) able to identify suitable potential participants provided them with information about the study. Potential participants who were interested were contacted by the research team to arrange a visit and obtain consent. Community control participants were recruited from local schools and youth groups. Details of the study were provided during recruitment. Participants were grouped by residential status (ie, community control participants or young people in care) and recent self-harm history (defined as the number of episodes of self-injury in the past year: no episodes, 1-4 episodes, or ≥5 episodes) made with mixed or unclear intent in line with the UK National Institute for Health and Care Excellence (NICE) guidelines.^30^ Young people with suicidal intent were not excluded. Exclusion criteria included having a known intellectual disability (intelligence quotient <70), autism spectrum disorder, heart or circulatory problems, epilepsy, or recent injury of a serious nature. Participants received a £50 gift voucher; the specific amount was withheld until consent was obtained to avoid inducement. Recruitment began in January 2019 and ended in March 2020. All procedures were performed in accordance with the Declaration of Helsinki.^63^

### Outcome Measures

The primary outcome measure was somatosensory sensitivity of participants who self-harm and live in residential care, which we compared with 2 control groups with no self-harm—control participants living in the community and control participants living in residential and/or group care. Information about individual participant’s sensitivity to a range of painful and nonpainful stimuli was obtained using the standardized quantitative sensory testing (QST) protocols developed by the German Research Network on Neuropathic Pain (DFNS)^31,32^ (eAppendix in the Supplement). The protocols measured 13 parameters to test for specific thermal and mechanical stimuli: cold detection thresholds (CDT), warm detection thresholds (WDT), thermal sensory limen (TSL), paradoxical heat sensations (PHS), cold pain thresholds (CPT), heat pain thresholds (HPT), mechanical detection threshold (MDT), mechanical pain threshold (MPT), mechanical pain sensitivity (MPS), wind-up ratio (WUR), dynamical mechanical allodynia (DMA), pressure pain threshold (PPT) and vibration detection threshold (VDT). PHS and DMA do not normally occur in healthy patients, and *z* transformation is not possible for these parameters, and therefore they were excluded from analysis.^33^ All tests were carried out on the volar forearm when possible, and areas of significant scarification were avoided in favor of naive skin. In some cases, the dorsal forearm or upper arm may have been used.

For clarity and ease of interpretation, each QST variable was *z* transformed using the appropriate age and gender group of published reference data from healthy control participants:

*z* score = (mean_participant_ – mean_reference data_) / SD_reference data_

The QST *z* score graph shows the direction of sensory change and whether or not the change is unusual. Positive *z* scores indicate gain of sensory function (ie, hypersensitivity) and negative *z* scores indicate loss of sensory function (ie, hyposensitivity). *z* scores greater than ±1.96 indicate values outside the 95% CI of the mean reference values and are considered unusual and potentially abnormal.

Our primary hypothesis was that the group with 5 or more self-harm episodes would show pain hyposensitivity compared with community-dwelling control participants. In addition to analyzing the painful (CPT, HPT, MPT, MPS, PPT) and nonpainful (CDT, WDT, TSL, MDT, VDT) QST variables separately, we also used a nonstandard approach to DFNS QST analysis to generate mean sensitivity scores for both painful and nonpainful items. The rationale was to reduce the number of comparisons in the analysis. The mean scores are the composite *z* scores across tasks and are presented as group-level means. Prescription drug use was a secondary outcome measure included in the analysis.

### Statistical Analysis

The QST variable WUR was excluded from analysis because 26 participants (control, n = 4; no self-harm [SH], n = 4; 1-4 SH episodes, n = 4; 5 or more SH episodes, n = 14) rated the single pinprick stimulus as 0 or not painful 3 or more times, therefore WUR could not be calculated. One male participant in the control group had his MDT score excluded from analysis because it was felt he did not perform the test correctly.

QST variables were compared between the 4 groups using linear regression with group as a dummy-coded independent variable followed by pairwise comparisons. Given that age, gender, and prescription drug use are factors that can influence QST responses,^31,32,34^ we adjusted for each of our regression analyses in a second step. Estimated marginal mean differences and 95% CI are presented following a significant overall *F* test for group for mean pain and sensory scores. For an explorative analysis of the ability to differentiate between groups of individual items, we performed a linear regression with group as the independent variable and estimated the explained variance for between-group differences.

To assess the validity of the mean scores, we performed pairwise Pearson correlations to measure how strongly the items were linearly associated with the relevant mean scores. We used principal component analysis to reduce the dimensionality of our 10 variables into a smaller set of variables (components) and to preserve as much variability as possible. The components are linear functions of the original variables, which permitted assessment of important patterns in the data. The principal components are interpreted by examining the magnitude and direction of coefficients of the original variables. We visually assessed the patterns of loadings (correlations) of the items on the first 2 components using a loading plot. Loadings close to +1 or −1 indicate which variables are strongly associated with the component. A high correlation between 2 items lead to 2 vectors that are very close to each other. If 2 vectors meet each other at 90°, the 2 items are not correlated, and if the vectors diverge and form a large angle that is closer to 180°, then the items are negatively correlated.

To assess whether mean pain and sensory scores can statistically estimate the incidence of self-harm within the previous year, we performed exploratory logistic regressions with self-harm as an outcome and mean pain and sensory score and 3 additional factors (ie, gender, age, and prescription drug use). We used 5-fold cross-validation (with 50 repeats to obtain stable results) to get nearly unbiased estimates of the accuracy of estimates of new unseen cases of the same population (internal validation).^35^ We used area under the curve (AUC) as overall discriminatory measures, which are independent of a threshold needed to predict class membership and prevalence of an outcome. AUC ranges from 0.5 (no discrimination) to 1 (perfect discrimination). According to Hosmer et al,^36^ AUC discrimination greater than 0.7 is classified as acceptable; greater than 0.8 as excellent, and greater than or equal to 0.9 as outstanding discrimination. Sensitivity, which is the ability of a test to correctly identify participants with self-harm, and specificity, which is the ability of a test to correctly identify participants with no self-harm, as additional measures. A cut-off value of *P* = .50 above which a test classifies class membership was used to calculate sensitivity and specificity.

In a second step, we performed explorative least absolute shrinkage and selection operator (LASSO) logistic regression, which performs automatic variable selection and penalizes (or regularizes) regression coefficients to reduce overfitting.^35^ Because the degree of regularization needs to be determined using cross-validation, we performed nested cross-validation (with 10 repeats of 5-fold for the additional outer loop) to get an unbiased estimate of accuracy.^37^ LASSO was also used to assess individual pain items instead of the mean pain score in combination with age, gender and prescription drug use. Cross-validation and LASSO regressions were done using R version 4.0 (R Project for Statistical Computing)^38^ and the user-written package glmnet^39^ and caret.^40^ All other analyses were done using Stata 16 (StataCorp).^41^ The 2-tailed α level for all statistical tests was set at .05.

The sample size was calculated using G*power v3.1.^42^ It has previously been shown^43^ that the difference in CPT between healthy adult control patients and young people in residential care with 5 or more incidences of self-harm within the previous year was approximately 6 °C (pooled SD = 8.1; control participants: 15.0 °C; self-harm: 8.9 °C, *d* = 0.74). Because we used age-matched control participants, we anticipated this difference to be slightly larger, and a sample size of 23 people per group was needed to have 80% power using an α of .05 and 2 tails to detect a difference of 7 °C assuming an SD of 8.1 (*d* = 0.86). To account for attrition, we allowed a larger sample size of 26 adolescents in each group (104 participants for 4 groups), allowing us to detect a difference of 6.5 °C for our primary hypotheses. Recruitment for the study ceased in March 2020 because of the COVID-19 pandemic.

## Results

A total of 64 participants ages 13 to 17 years completed testing (mean [SD] age, 16.3 [1.0] years; 34 [53%] females and 30 [47%] males; 50 [78%] living in group homes). Of the total, 14 participants were in the control group (mean [SD] age, 16.4 [0.7] years; 12 [86%] females, 2 [14%] males); 17 participants were in the no SH group (mean [SD] age, 16.5 [1.0] years; 1 [6%] female, 16 [94%] males); 12 participants were in the SH group with 1 to 4 episodes (mean [SD] age, 16.2 [1.4] years; 4 [33%] females, 8 [66%] males); and 21 participants were in the SH group with 5 or more episodes (mean [SD] age, 16.3 [1] year; 17 [81%] females, 4 [19%] males). Participant characteristics are summarized in Table 1.

**Table 1.** Participant Characteristics (N = 64)

Characteristic	No. (%)			
	Control^a^	No SH	SH 1-4	SH ≥5
No.	14	17	12	21
Age, mean (SD), y	16.4 (0.67)	16.5 (1.02)	16.2 (1.39)	16.3 (0.99)
Gender				
Male	2 (14.3)	16 (94.1)	8 (66.7)	4 (19)
Female	12 (85.7)	1 (5.9)	4 (33.3)	17 (81)
Ethnicity				
White British	11 (78.6)	15 (88.2)	11 (91.7)	18 (85.7)
Ethnic minority^b^	3 (21.4)	2 (11.8)	1 (8.3)	3 (14.3)
Medication				
None	12 (85.7)	14 (82.4)	5 (41.7)	8 (38.1)
Antidepressant	2 (14.2)	1 (5.9)	4 (33.3)	3 (14.3)
Antipsychotic	1 (7.1)	2 (11.8)	1 (8.3)	2 (9.5)
Other	0	3 (17.7)	5 (41.7)	13 (61.9)
Diagnosis^c^				
None	14 (100)	6 (35.3)	1 (8.3)	1 (4.8)
Internalizing	0	4 (23.5)	5 41.7)	18 (85.7)
Externalizing	0	8 (47.1)	5 (41.7)	0
Neurodevelopmental	0	1 (5.9)	3 (25)	4 (19.1)
BPD threshold^d^	0	1 (5.9)	7 (58.3)	14 (66.7)
Mean (SD)	3 (2.22)	3.06 (2.28)	6.64 (2.25)	6.76 (2.63)
Suicidal thinking prior 6 mos	3 (21.4)	0 (0)	2 (16.7)	12 (57.1)

Abbreviations: BPD, borderline personality disorder; SH, episodes of self-harm within the previous year.

^a^ Community control group included 3 participants with history of self-harm but not within previous year.

^b^ Ethnic minority describes individuals self-identifying as any ethnic group except the White British group.

^c^ Diagnosis included internalizing (ie, anxiety, depression, and mood disorders); externalizing (ie, conduct and substance disorders); neurodevelopmental (ie, attention deficit hyperactivity disorder).

^d^ BPD threshold was collected using the McLean Screening Instrument. The range of possible scores is 0-10; scores greater than or equal to 7 meet criteria for BPD.

### Pain Sensitivity and Frequency of Self-harm

**Figure 1.**
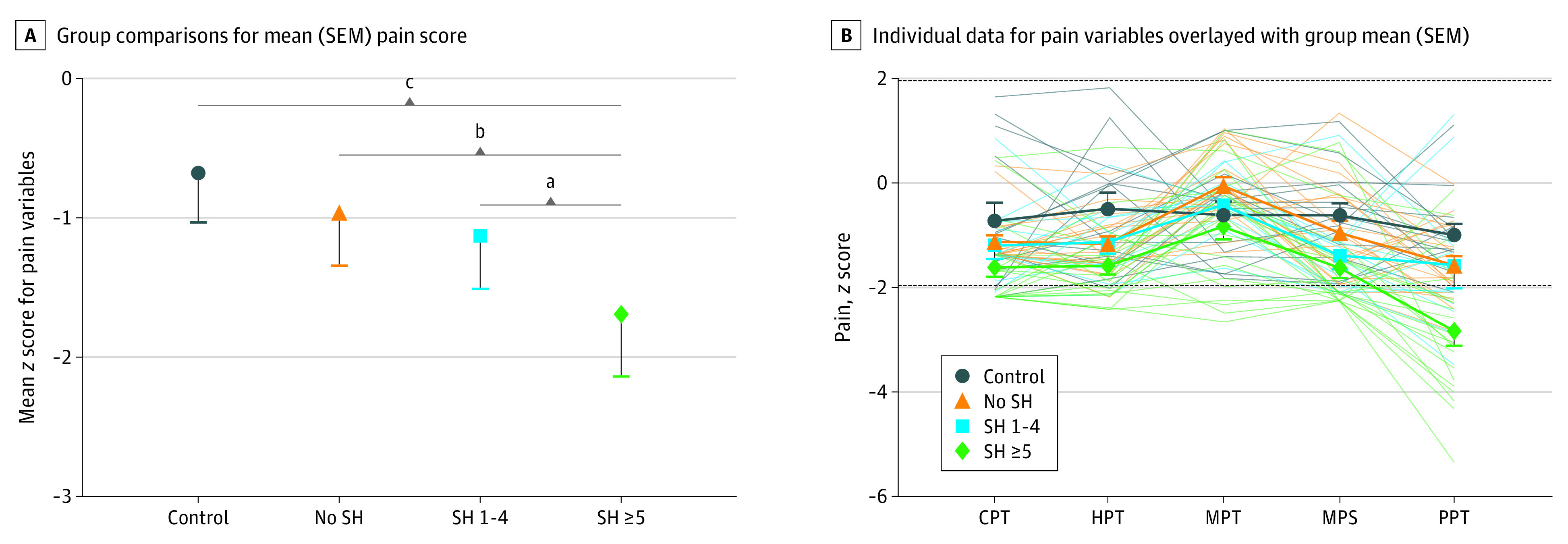
Quantitative Sensory Testing (QST) *z *Scores for Unadjusted Pain Parameters Each parameter shows significant variation by group and a similar trend to the group mean (SEM) pain scores, with the most frequent self-harm group showing significant hyposensitivity. Results outside of the SD of 1.96 (dotted line) indicate potentially abnormal thresholds. CPT indicates cold pain threshold; HPT, heat pain threshold; MPT, mechanical pain threshold; MPS, mechanical pain sensitivity; PPT, pressure pain threshold; SH, episodes of self-harm within the previous year; SH 1-4, 1 to 4 self-harm episodes within the previous year; SH ≥5, 5 or more self-harm episodes within the previous year. ^a^*P* < .05. ^b^*P* < .001. ^c^*P* < .01.

We used 5 different pain sensitivity tests and calculated a mean pain score for each of the 4 groups. All the individual pain measures were highly and significantly correlated with the mean pain score (Pearson *r* > 0.65 in all cases; *P* < .001) (eTable 1 in the Supplement). We compared the mean pain score between the 4 groups and found pain sensitivity to be significantly lower in the high self-harm group compared with both control groups (unadjusted: SH group with 5 or more episodes vs control, −1.01 [95% CI, −1.45 to −0.57]; *P* < .001; SH group with 5 or more episodes vs no SH, −0.73 [95% CI, −1.14 to −0.31]; *P* = .001) (Figure 1 and Table 2). The SH group with 1 to 4 episodes had an intermediate phenotype and were significantly different from the SH group with 5 or more episodes (mean [SEM], −0.56 [95% CI, −1.02 to −0.11]; *P* = .02) but did not reach significance compared with the no SH control groups. After adjusting for age, gender, and prescription drug use, the SH group with 5 or more episodes remained significantly different from the community control group but were not significantly different from the no SH or SH group with 1 to 4 episodes, and a significant difference was seen between the SH group with 1 to 4 episodes and control groups, which was not significant when the data were unadjusted (SH group with 5 or more episodes vs control, mean [SEM], −1.03 [95% CI, −1.47 to −0.60]; *P* < .001; SH group with 1 to 4 episodes vs control, −0.65 [95% CI, −1.19 to −0.1]; *P* = .02) (Table 2). Individual pain parameters are presented as *z *scores, allowing direct comparison of the different measures, and pairwise comparisons for the individual pain tests reflect the trend observed with the mean pain score group comparisons (Figure 1 and eTable 2 in the Supplement). Not all measures showed equal magnitude of change, with MPT showing, on average, the least difference in participants with self-harm and PPT showing the greatest.

**Table 2.** Pairwise Group Comparisons for Mean Pain Score (95% CI) Unadjusted and Adjusted for Age, Gender, and Prescription Drug Use

Pairwise comparison	Mean difference (95% CI)	*t*	*P* value
**Pain**			
Group only	*F*_3,60_ = 8.09	*R*^2^, 0.29 (95% CI, 0.09 to 0.43)	<.001
No SH vs control	−0.28 (−0.74 to 0.18)	−1.22	.23
SH 1-4 vs control	−0.44 (−0.94 to 0.06)	−1.78	.08
SH ≥5 vs control	−1.01 (−1.45 to −0.57)	−4.60	<.001
SH 1-4 vs no SH	−0.17 (−0.64 to 0.31)	−0.69	.49
SH ≥5 vs no SH	−0.73 (−1.14 to −0.31)	−3.52	.001
SH ≥5 vs SH 1-4	−0.56 (−1.02 to −0.11)	−2.46	.02
Adjusted for age, gender and prescription drug use	*F*_5,58 _= 5.58	*R*^2^, 0.34, (95% CI, 0.09 to 0.45)	<.001
No SH vs control	−0.57 (−1.14 to 0.002)	−1.99	.05
SH 1-4 vs control	−0.65 (−1.19 to −0.1)	−2.37	.02
SH ≥5 vs control	−1.03 (−1.47 to −0.60)	−4.75	<.001
SH 1-4 vs no SH	−0.08 (−0.57 to 0.41)	−0.32	.75
SH ≥5 vs no SH	−0.47 (−0.99 to 0.06)	−1.78	.08
SH ≥5 vs SH 1-4	−0.39 (−0.89 to 0.11)	−1.55	.13

Abbreviations: *R*^2^, variance; SH, episodes of self-harm within the previous year.

Next, we compared the proportion of the variance (*r*^2^) as a measure of effect size for each pain test that is attributable to group differences. Except for MPT (10.7% [95% CI, 0-23.2%] *F* test, *P* = .08), group differences explained significant proportions of variance: CPT, 12.5% (95% CI, 0.0%-25.5%); *P* = .04; HPT, 19.9% (95% CI, 2.6%-33.8%); *P* = .01; MPS, 16.0% (95% CI, 0.6%-29.5%); *P* = .02; PPT, 31.1% (95% CI, 10.5%-44.7%); *P* < .001. PPT showed the strongest correlation with the mean pain score (Pearson *r* = 0.81).

### Responses to Nonpainful Sensory Tests 

**Figure 2.**
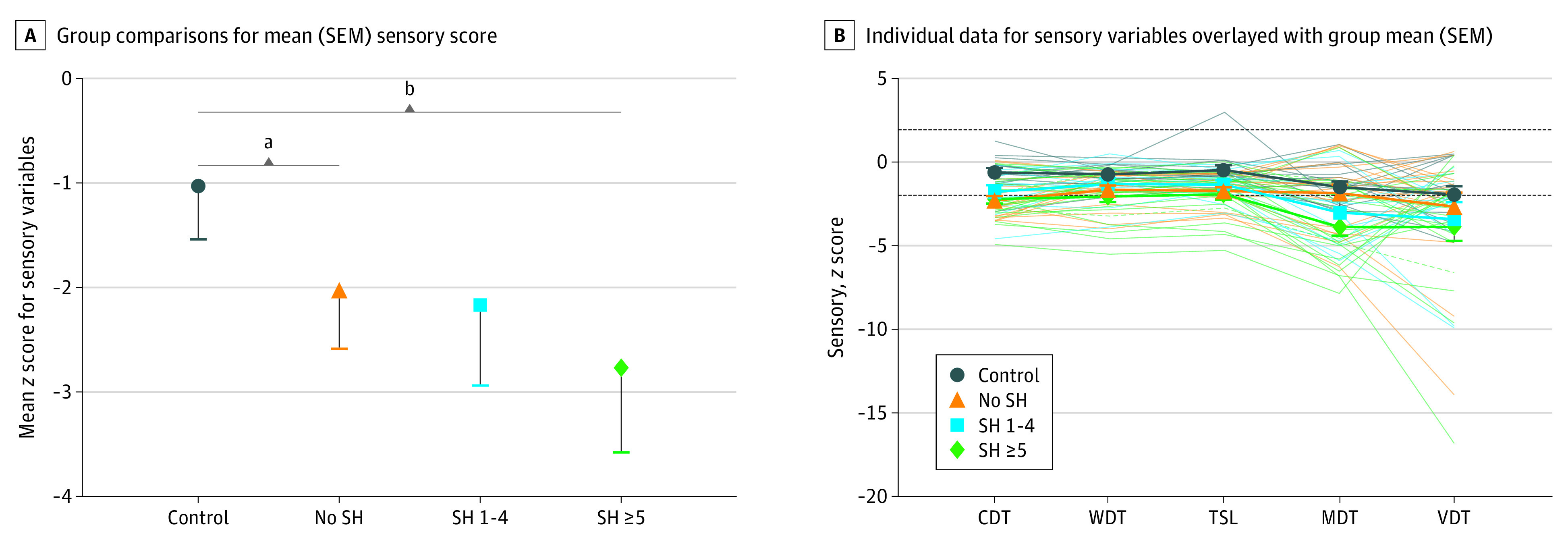
Quantitative Sensory Testing (QST) *z *Scores for Sensory (Nonpain) Parameters (Unadjusted) The SH ≥5 group show significant hyposensitivity to sensory stimuli in general, and the young people in residential care with no SH show significant variation in thermal sensitivity compared with community control participants. Results outside of the SD of 1.96 (dotted line) indicate potentially abnormal thresholds. CDT indicates cold detection threshold; WDT, warm detection threshold; TSL, thermal sensory limen; MDT, mechanical detection threshold; SH, episodes of self-harm within the previous year; SH 1-4, 1 to 4 self-harm episodes within the previous year; SH ≥5, 5 or more self-harm episodes within the previous year; VDT, vibration detection threshold. ^a^*P* < .05. ^b^*P* < .001.

We also tested the participants for a variety of nonpainful sensory measures using thermal and tactile stimuli, and individual scores were used to generate a mean nonpain sensory score. All individual sensory items were associated with the mean sensory score (eTable 3 in the Supplement). The mean sensory score was significantly reduced in the most frequent self-harm group compared with community control participants (SH ≥5 vs control, −1.75 [95% CI, −2.62 to −0.88]; *P *< .001) (Figure 2 and eTable 2 in the Supplement). Surprisingly, we also found a significant difference between the community control participants and the young people in residential care with no self-harm (mean [SEM], −1.09; [95% CI = −2.0 to −0.18]; *P* = .02). The latter difference became nonsignificant after adjusting for age, gender, and prescription drug use, but the overall patterns remained the same. Individual sensory parameters are presented as *z* scores with pairwise comparisons for the individual tests (Figure 2 and eTable 2 in the Supplement). We found highly variable responses to the VDT test for all groups but observed a similar dose-dependent pattern of response to nonpainful stimuli as that seen with painful stimuli. For example, the most frequent self-harm group showed the greatest sensory deficit compared with the other groups tested (Table 3). Following adjustment for age, gender, and prescription drug use, only the difference between SH ≥5 vs control remained significant (−1.73 [95% CI, −2.62 to −0.84]; *P* < .001).

**Table 3.** Pairwise Group Comparisons for Mean Sensory Score (95% CI) Unadjusted and Adjusted for Age, Gender, and Prescription Drug Use

Pairwise comparison	Mean difference (95% CI)	*t*	*P* value
Group only	*F*_3,60_ = 5.40	*R*^2^ = 0.21 (95% CI, 0.03 to 0.35)	.002
No SH vs control	−1.09 (−2.01 to −0.18)	−2.4	.02
SH 1-4 vs control	−0.99 (−1.98 to 0.003)	−2	.05
SH ≥5 vs control	−1.75 (−2.62 to −0.88)	−4.02	<.001
SH 1-4 vs no SH	0.10 (−0.85 to 1.06)	0.22	.83
SH ≥5 vs no SH	−0.66 (−1.48 to 0.17)	−1.6	.12
SH ≥5 vs SH 1-4	−0.76 (−1.67 to 0.15)	−1.66	.10
Adjusted for age, gender, and antidepressant	*F*_6,57_ = 5.24	*R*^2^ = 0.24 (95% CI, 0.01 to 0.34)	.003
No SH vs control	−0.913 (−2.07 to 0.25)	−1.58	.12
SH 1-4 vs control	−0.86 (−1.96 to 0.25)	−1.54	.13
SH ≥5 vs control	−1.73 (−2.62 to −0.84)	−3.91	<.001
SH 1-4 vs no SH	0.06 (−0.94 to 1.06)	0.12	.91
SH ≥5 vs no SH	−0.82 (−1.88 to 0.25)	−1.54	.13
SH ≥5 vs SH 1-4	−0.88 (−1.89 to 0.14)	−1.72	.09

Abbreviation: SH, episodes of self-harm within the previous year.

### Estimation of Self-harm

At the group level, the discrimination between pain and nonpain sensory tests was further supported by principal component analysis of all the sensory variables studied here. The first 2 components identified accounted for 58% of the variance and dissociated the pain from nonpain sensory tests (eFigure in the Supplement). We undertook exploratory logistic regression analysis to identify which variables were important for the statistical estimation of self-harm within the last year and whether the mean pain score can be replaced with an individual pain item. A model that included mean pain and nonpain sensory scores as well as age, gender, and prescription drug use had a cross-validated AUC of 0.79 (sensitivity, 0.70; specificity, 0.80). Omitting mean sensory score resulted in a slightly better model (cross-validated AUC, 0.80; sensitivity, 0.72; specificity, 0.77), suggesting that mean sensory score is not necessary for prediction. Rerunning the model as a LASSO regression to reduce overfitting and automatic variable selection of redundant variables suggests that all 4 variables (ie, mean pain score, age, gender, and prescription drug use) are important. Nested cross-validation to correct for model selection resulted in an AUC of 0.79 (sensitivity, 0.76; specificity, 0.70). However, age, gender, and prescription drug use alone performed less well (AUC, 0.72; sensitivity, 0.78; specificity, 0.59), while mean pain score alone performed reasonably well (AUC, 0.77; sensitivity, 0.85; specificity, 0.39). Reasonable clinical implementation, accounting for the cost of equipment and ease of use, suggests that PPT (AUC, 0.76; sensitivity, 0.72; specificity, 0.61) offers the best solution, as it is quick to perform (<1 minute), inexpensive, and easy to interpret. Models and resulting estimation accuracy as the area under the receiver operating characteristic curve are shown in eTable 4 in the Supplement.

## Discussion

Our findings suggest that reduced pain sensitivity, evident in response to a broad range of painful stimuli, could be a phenotype of adolescents with self-harm. Individual pain QST scores were used to generate a composite mean pain score, which also showed the same result after controlling for age, gender, and prescription drug use. These findings are novel evidence that these sensory differences also extend to nonpainful stimuli, and adolescents with the most frequent episodes of self-harm show the largest mean nonpain sensory deficit. Finally, we systematically examined which of the tests accounted for a significant amount of variance in the data. For routine evaluation of risk, a single QST measure would be more convenient, and PPT, which is simple, quick, and inexpensive to implement, offers the best clinical option from those tested here (AUC: 0.76). PPT was the most distinguished clinical test between the 4 participant groups (31.1%; *P* < .001) and showed the strongest correlation with the mean pain score (Pearson *r* = 0.81) (eTable 1 in the Supplement). Our findings suggest that reduced pain sensitivity is associated with self-harm in adolescents.

Consistent with some previous studies, we report hyposensitivity to pressure pain in adolescents with self-harm.^24^ We extended this by demonstrating a dose-dependent pain hyposensitivity that increased with the incidence of self-harm across a range of stimuli. This supports Joiner’s^19,44^ interpersonal theory of suicidal behavior which suggests that repeated self-harm behavior leads to habituation of painful stimuli and a reduced fear of pain. The comprehensive range of tests used in this study likely enhanced our sensitivity to detect sensory differences.^31,34,45^ A notable feature of this analysis was the consistency of changes, meaning all the different QST pain measures varied with the incidence of self-harm. However, this is not the case in many other pathologies associated with altered pain sensations, such as various forms of neuropathy.^46^

We found that nonpain sensory tests did not discriminate self-harm but did reveal interesting differences between adolescents living at home vs those with history of abuse and maltreatment living in residential care settings. Young people in residential care with no self-harm were associated with significantly decreased sensitivity to nonpainful sensory stimuli compared with community control participants. Despite nonpainful sensory changes not showing the same association with self-harm frequency as the pain tests, we observed a strong and consistent reduction in sensitivity to nonpainful stimuli in all young people in residential care compared with community control participants. Pain hyposensitivity might serve as a risk factor for self-harm behavior rather than be a result of self-harm behavior.^24^ Our finding that adolescents in residential care have sensory abnormalities regardless of incidence of self-harm is consistent with this assertion. Importantly, our cohort of adolescents with self-harm almost entirely comprised young people living in residential care. Childhood maltreatment is not only a risk factor for self-harm but also dissociation, which is a risk-factor for self-harm, and both have been previously shown to affect pain perception.^44,47,48,49,50^ These sensory changes are unlikely to represent a physiological difference in the processing of noxious information by the peripheral nervous system. Rather it is highly probable that alterations in pain perception are reflective of central nervous system changes in the functionality of the descending pain modulatory pathways.^51^

### Limitations

This study has limitations. The novel features reported here are made without an attempt to disentangle subjective response and the neurobiology of physical pain or general somatosensory sensitivity. Although our sample was consisted of both male and female adolescents, genders were not evenly distributed within our groups, and therefore, we were unable to investigate gender differences directly. Girls made up most of the community control group and the self-harm group with 5 or more episodes, whereas boys were the majority among the young people in residential care with no self-harm. Our results are also likely biased because we did not have a comparison group of community-dwelling adolescents with self-harm. There is much evidence to suggest that childhood maltreatment results in psychobiological changes,^52,53,54,55,56,57,58^ and our study lacked the sensitivity to examine this in-depth. Additionally, previous systematic meta-analysis has found that major depression is associated with increased physical pain thresholds (ie, hyposensitivity), which we did not account for in our analysis.^59^ However, these findings were based on older cohorts with a mean age of 35 years so we cannot discount other life events or age-related changes impacting on these results. Importantly, our findings align with previous studies that have found PPT to be elevated in patients with suicide ideation and suicide attempts.^60^ Self-harm is one of the strongest known factors of suicide. We did not account for occasional use of NSAIDs or other recreational drug use, but we did account for prescription medication (eg, SSRIs), which are known to affect pain perception. We did not assess for chronic pain. Finally, although we used cross-validation to account for overfitting, we did not correct for comparing a set of models and our best model may be optimistic. Although penalization methods will generally improve on standard estimation methods, studies with small sample sizes can produce unreliable data sets.^61^ Because of the limited sample size, we did not assess calibration.^62^ The large number of explorative tests and estimation models limits the reproducibility and generalizability of our findings and results; therefore, overall patterns should be given more emphasis than individual tests.

## Conclusions

In this study, pain sensitivity appeared to be a biomarker for incidence and frequency of self-harm, and we propose PPT as a measure of pain sensitivity to assess the risk of self-harm in adolescents. How pain sensitivity changes with age and whether these sensory changes remit with changing incidence or cessation of self-harm over time is unknown. Future studies should explore whether a test of pain sensitivity can predict the onset of self-harm and completed suicides. Furthermore, these findings could be extended to longitudinal research in adults who currently or previously had self-harmed to examine whether sensory hyposensitivity in adolescents is lost and how sensory sensitivity manifests in adulthood.

## Notes

Supplement. **eAppendix.** Description of Quantitative Sensory Tests (QST) Used in the Study **eTable 1.** Pearson Correlation Matrix of Individual Pain Scores and Mean Pain Score **eTable 2.** Pain Score and Sensory Score Pairwise Comparisons **eTable 3.** Pearson Correlation Matrix of Individual Nonpain Sensory Scores and Mean Sensory Score **eFigure.** Scatterplot of Loadings (Components) After Principal Component Analysis of the 4 Sensory (CDT, WDT, TSL, MDT) and 5 Pain Items (CPT, HPT, MPT, MPS, PPT) **eTable 4.** Models and Prediction Accuracy (Sensitivity/Specificity) for Incidence of Self-harm Within the Past Year
