# Supplementary material for: Assessment of Somatosensory Function and Self-harm in Adolescents
Source: JAMA Netw Open. 2021 Jul 13;4(7):e2116853. doi: 10.1001/jamanetworkopen.2021.16853 (PMC8278268; doi:10.1001/jamanetworkopen.2021.16853)
Supplement: Supplement. — eAppendix. Description of Quantitative Sensory Tests (QST) Used in the Study eTable 1. Pearson Correlation Matrix of Individual Pain Scores and Mean Pain Score eTable 2. Pain Score and Sensory Score Pairwise Comparisons eTable 3. Pearson Correlation Matrix of Individual Nonpain Sensory Scores and Mean Sensory Score eFigure. Scatterplot of Loadings (Components) After Principal Component Analysis of the 4 Sensory (CDT, WDT, TSL, MDT) and 5 Pain Items (CPT, HPT, MPT, MPS, PPT) eTable 4. Models and Prediction Accuracy (Sensitivity/Specificity) for Incidence of Self-harm Within the Past Year [file jamanetwopen-e2116853-s001.pdf]

Supplemental Online Content

Cummins TM, English O, Minnis H, et al. Assessment of somatosensory function and self-harm in adolescents. *JAMA Netw Open*. 2021;4(7):e2116853.
doi:10.1001/jamanetworkopen.2021.16853

eAppendix. Description of Quantitative Sensory Tests (QST) Used in the Study

eTable 1. Pearson Correlation Matrix of Individual Pain Scores and Mean Pain Score

eTable 2. Pain Score and Sensory Score Pairwise Comparisons

eTable 3. Pearson Correlation Matrix of Individual Nonpain Sensory Scores and Mean Sensory Score

eFigure. Scatterplot of Loadings (Components) After Principal Component Analysis of the 4 Sensory (CDT, WDT, TSL, MDT) and 5 Pain Items (CPT, HPT, MPT, MPS, PPT)

eTable 4. Models and Prediction Accuracy (Sensitivity/Specificity) for Incidence of Self-harm Within the Past Year

This supplemental material has been provided by the authors to give readers additional information about their work.

© 2021 Cummins TM et al. *JAMA Network Open.*

eAppendix. Description of quantitative sensory tests (QST) used in the study.

The DFNS QST protocol measures cutaneous sensitivity to a range of stimulus modalities including temperature, light touch, strong pressure and vibration. The tests provide a complete sensory profile for one region of skin within 30 minutes while the participant is seated in a comfortable chair. The purpose is to provide insight into peripheral- and central pain and touch processing mechanisms via subjective reporting of stimulus detection and pain thresholds. The protocol is highly standardised allowing data to be compared across different study populations. The DFNS has published skin-region specific, normative reference values from a large cohort of healthy controls stratified by age and sex. Individual data are z-transformed using reference values to determine whether individual data are within the range of normal variability. For full details on the reference values, including range of possible scores in their original units, and z-transformation methods we refer the interested reader to Magerl et al., (2010)[1] and Supplementary Table 1 within.

Thermal detection and pain thresholds were tested using the computerised TSA-II NeuroSensory Analyser (Medoc, Israel). Water was circulated through a 3.2 cm2 thermode (a Peltier device) secured against the forearm by a Velcro strap. Temperature change was programmed to occur from a baseline temperature of 32 °C, with heating and cooling delivered at a rate of 1 °C/sec to a maximum of 50 °C and 0 °C, respectively (interstimulus interval: 6 sec; baseline return rate: 8 °C/sec). Each threshold test was performed three times. For assessment of cold and warm detection thresholds (CDT and WDT), participants were asked to press a stop button as soon as they noticed a change in temperature above or below baseline temperature. The arithmetic means for both CDT and WDT were calculated using the difference between the measured threshold temperature and the baseline temperature (32 °C). Following, the thermal sensory limen (TSL) was assessed which is a detection threshold test using alternating warming and cooling temperatures (six temperature changes in total) to assess paradoxical heat sensations (PHS). Participants pushed a stop button when they noticed a change in temperature from baseline and reported the temperature sensation (e.g., 'warm'/'cold'/'burning'). The TSL was calculated by subtracting the arithmetic mean of the CDT (three measurements) from that of the WDT (three measurements). For assessment of cold and heat pain thresholds (CPT and HPT), participants were asked to press a stop button at their first pain sensation during warming and cooling of the thermode. The arithmetic mean of three repetitions was calculated using the absolute temperature values (°C) for CPT and HPT.

The mechanical detection threshold (MDT) was assessed using a set of standardised von Frey hairs (a pen-like instrument with a nylon thread attached; Optihair, Marstock Nervtest, Germany) ranging from 0.25 - 256 mN. Using a modified method of limits protocol, the hairs were lightly touched against the skin with increasing and decreasing intensity, and the participant was asked to indicate whether they detected the touch sensation. The test was performed in an 'up-down' pattern until five supra- and five subthreshold responses were taken. The geometric mean was calculated as the MDT. The mechanical pain threshold (MPT) was performed using weighted pinprick stimuli (MRC Systems GmbH, Germany) ranging from 8 – 512 mN. The pinprick tips have a flat contact area of 0.25mm in diameter. Like MDT, the test was performed in an 'up-down' pattern until five supra- and five subthreshold responses were taken. The geometric mean was calculated as the MPT.

Mechanical pain sensitivity (MPS) and dynamic mechanical allodynia (DMA) (stimulus–response function model) were assessed using a combination of light-touch stimuli (cotton wool, a soft brush and a cotton bud) and pinprick stimulators touched against the skin in a pseudorandom order. Participants were asked to rate the painfulness of each stimulation using a numeral rating scale anchored at 0 (no pain) and 100 (worst pain imaginable). A total of 50 stimulations were given and pain sensitivity was calculated by the geometric mean of the pain ratings given for pinprick stimuli (MPS) and innocuous stimuli (DMA) separately.

Wind-up ratio (WUR) was assessed using a 256 mN pinprick stimulator. Participants were asked to rate the painfulness of a single stimulation (numerical rating scale 0-100). This was followed shortly after by a short train of 10 stimuli (repetition rate of 1/sec). At the end of the train, participants were asked to provide a second pain rating. This was repeated five times in a (non-overlapping) small region of skin. For each of the five test repetitions, the arithmetic mean ratio was calculated by dividing the pain rating for the stimulus train with that of the single stimulus. The geometric mean was calculated from these ratios to generate a WUR score. If a rating of '0' was given three times for the single stimulus (i.e., three of the five repetitions) the test was not continued as WUR could not be calculated.

Vibration detection threshold (VDT) was assessed using an activated 64 Hz tuning fork (Ragg, Sheffield, UK) which was placed on the bony prominence at the wrist. The VDT was determined from the "wandering" tip of a

© 2021 Cummins TM et al. *JAMA Network Open.*

triangle indicated along a scale on the fork and moved by means of the vibration. Participants were asked to indicate the point at which they no longer perceived the vibration sensation. The test was performed three times and the arithmetic mean was calculated.

Pressure pain threshold (PPT) was assessed using a manual pressure algometer (Wagner Instruments, USA) pressed into the muscle at the base of the thumb. Pressure was increased at 0.5 kg/sec and participants were asked to say stop once the pressure first became painful. The test was repeated three times and the arithmetic mean (in kPa) was calculated as threshold.

1. Magerl W, Krumova EK, Baron R, Tolle T, Treede RD, Maier C. Reference data for quantitative sensory testing (QST): refined stratification for age and a novel method for statistical comparison of group data. *Pain.* 2010;151(3):598-605.

eTable 1. Pearson's correlation matrix of individual pain scores and mean pain score

Parameter	CPT	HPT	MPT	MPS	PPT	*M* Pain Score
**CPT**	1					
**HPT**	0.563	1				
**MPT**	0.301	0.224	1			
**MPS**	0.297	0.362	0.507	1		
**PPT**	0.516	0.527	0.367	0.375	1	
***M* Pain Score**	0.730	0.727	0.652	0.693	0.807	1

Abbreviations: CPT, cold pain threshold; HPT, heat pain threshold; MPT, mechanical pain threshold; MPS, mechanical pain sensitivity; PPT, pressure pain threshold

© 2021 Cummins TM et al. *JAMA Network Open.*

eTable 2. Pain Score and Sensory Score Pairwise Comparisons

Pain Score		
**Pain parameter**	**Factor Group *P***	**Group**
**CPT**	0.006	SH 5+ < Control
	<0.001	SH 5+ < Control
	0.023	No SH < Control
**HPT**		SH 1-4 < Control (*P* = 0.062)
**MPT**	0.012	SH 5+ < No SH
	0.003	SH 5+ < Control
	0.030	SH 5+ < No SH
**MPS**	0.048	SH 1-4 < Control
	<0.001	SH 5+ < Control
	0.001	SH 5+ < No SH
**PPT**	0.004	SH 5+ < SH 1-4
**Sensory Score**		
**Sensory parameter**	**Factor Group *P***	**Group**
	<0.001	SH 5+ < Control
	<0.001	No SH < Control
	0.031	SH 1-4 < Control
**CDT**		SH 1-4 vs No SH (*P* = 0.067)
**WDT**	0.002	SH 5+ < Control
	0.023	No SH < Control
		SH 5+ < SH 1-4 (*P* = 0.058)
	<0.001	SH 5+ < Control
	0.003	No SH < Control
**TSL**		SH 1-4 < Control (*P* = 0.060)
	0.002	SH 5+ < Control
**MDT**	0.005	SH 5+ < No SH
**VDT**		No follow-up

© 2021 Cummins TM et al. *JAMA Network Open.*

eTable 3. Pearson's Correlation Matrix of Individual Nonpain Sensory Scores and Mean Sensory Score

Parameter	CDT	WDT	TSL	MDT	VDT	*M* Sensory Score
**CDT**	1					
**WDT**	0.722	1				
**TSL**	0.661	0.871	1			
**MDT**	0.463	0.694	0.614	1		
**VDT**	0.121	0.161	0.137	0.377	1	
***M* Sensory Score**	0.650	0.780	0.730	0.836	0.685	1

Abbreviations : CDT, cold detection threshold; WDT, warm detection threshold; TSL, thermal sensory limen; MDT, mechanical detection threshold; VDT, vibration detection threshold

© 2021 Cummins TM et al. *JAMA Network Open.*

eFigure 1. Scatterplot of loadings (components) after principal component analysis of the five sensory items (CDT, WDT, TSL, MDT, VDT) and five pain items (CPT, HPT, MPT, MPS, PPT).

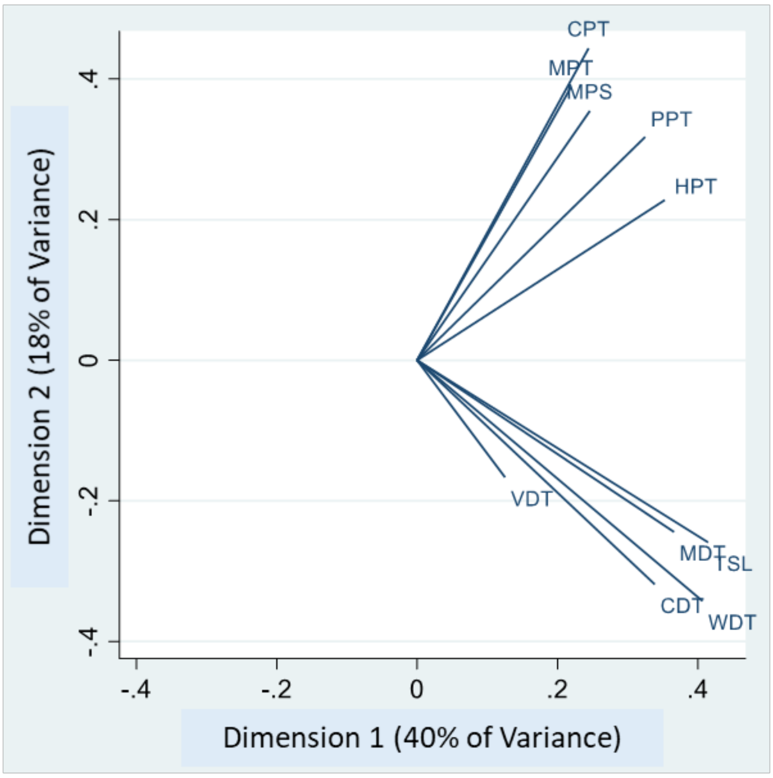

The loadings plot shows the relationship between the loadings of first two principal components and the original variables. Variables which are close (forming a small angle) are positively correlated. If they meet each other at 90 degrees are not correlated and if they form a large angle (close to 180) they are negatively correlated. The graph shows that the sensory items form one cluster of correlated items, mainly loading on component 1, and the pain items a second cluster, mainly loading at component 2. CPT, cold pain threshold; HPT, heat pain threshold; MPT, mechanical pain threshold; MPS, mechanical pain sensitivity; PPT, pressure pain threshold; CDT, cold detection threshold; WDT, warm detection threshold; TSL, thermal sensory limen; MDT, mechanical detection threshold; VDT, vibration detection threshold.

© 2021 Cummins TM et al. *JAMA Network Open.*

eTable 4. Models and Prediction Accuracy (Sensitivity/Specificity) for Incidence of Self-harm Within the Past Year

Predictors	Additional predictors	AUC	Sensitivity	Specificity
Mean pain and sensory scores	Age, Gender, Anti-Dep	0.80	0.79	0.71
Mean pain score	Age, Gender, Anti-Dep	0.79	0.76	0.70
Mean pain score	-	0.77	0.85	0.39
CPT	Age, Gender, Anti-Dep	0.75	0.65	0.69
HPT	Age, Gender, Anti-Dep	0.78	0.78	0.70
MPT	Age, Gender, Anti-Dep	0.73	0.78	0.61
MPS	Age, Gender, Anti-Dep	0.79	0.75	0.66
PPT	Age, Gender, Anti-Dep	0.76	0.72	0.61
-	Age, Gender, Anti-Dep	0.72	0.78	0.59

AUC, area under the curve; CPT, cold pain threshold; HPT, heat pain threshold; MPT, mechanical pain threshold; MPS, mechanical pain sensitivity; PPT, pressure pain threshold

© 2021 Cummins TM et al. *JAMA Network Open.*
